# Entropic Statistical Description of Big Data Quality in Hotel Customer Relationship Management

**DOI:** 10.3390/e21040419

**Published:** 2019-04-19

**Authors:** Lydia González-Serrano, Pilar Talón-Ballestero, Sergio Muñoz-Romero, Cristina Soguero-Ruiz, José Luis Rojo-Álvarez

**Affiliations:** 1 Department of Business and Management, Rey Juan Carlos University, 28943 Madrid, Spain; 2 Department of Theory and Comunications, Telematics and Computing Systems, Rey Juan Carlos University, 28943 Madrid, Spain

**Keywords:** Customer Relationship Management, hospitality industry, big data, duplicate detection, name matching, Levenshtein distance, *X-from-M* strategy, entropy, mutual information, mass density function

## Abstract

Customer Relationship Management (CRM) is a fundamental tool in the hospitality industry nowadays, which can be seen as a big-data scenario due to the large amount of recordings which are annually handled by managers. Data quality is crucial for the success of these systems, and one of the main issues to be solved by businesses in general and by hospitality businesses in particular in this setting is the identification of duplicated customers, which has not received much attention in recent literature, probably and partly because it is not an easy-to-state problem in statistical terms. In the present work, we address the problem statement of duplicated customer identification as a large-scale data analysis, and we propose and benchmark a general-purpose solution for it. Our system consists of four basic elements: (a) A generic feature representation for the customer fields in a simple table-shape database; (b) An efficient distance for comparison among feature values, in terms of the Wagner-Fischer algorithm to calculate the Levenshtein distance; (c) A big-data implementation using basic map-reduce techniques to readily support the comparison of strategies; (d) An *X-from-M* criterion to identify those possible neighbors to a duplicated-customer candidate. We analyze the mass density function of the distances in the CRM text-based fields and characterized their behavior and consistency in terms of the entropy and of the mutual information for these fields. Our experiments in a large CRM from a multinational hospitality chain show that the distance distributions are statistically consistent for each feature, and that neighbourhood thresholds are automatically adjusted by the system at a first step and they can be subsequently more-finely tuned according to the manager experience. The entropy distributions for the different variables, as well as the mutual information between pairs, are characterized by multimodal profiles, where a wide gap between close and far fields is often present. This motivates the proposal of the so-called *X-from-M* strategy, which is shown to be computationally affordable, and can provide the expert with a reduced number of duplicated candidates to supervise, with low *X* values being enough to warrant the sensitivity required at the automatic detection stage. The proposed system again encourages and supports the benefits of big-data technologies in CRM scenarios for hotel chains, and rather than the use of ad-hoc heuristic rules, it promotes the research and development of theoretically principled approaches.

## 1. Introduction

Customer Relationship Management (CRM) is a customer-centric business strategy which a company employs to improve customer experience and satisfaction by customizing products and services to their customers’ needs [1]. CRM systems offer companies the possibility of obtaining competitive advantages through better knowledge while maintaining a close relationship with their customers [2,3]. For the hospitality sector, CRM is considered as one of the best strategies to improve a company’s results and to ensure its long-term survival [4,5,6,7,8]. Accordingly, CRM systems are nowadays a fundamental tool in this sector, especially when properly implemented, as there is a large amount of customer data that are integrated by hotels today. These data can be turned into useful knowledge [9,10,11,12,13,14] to improve customer satisfaction and retention [15]. Furthermore, the growing importance of CRM systems has led to paying greater attention to the value of customer data as a strategic asset [16]. However, some studies [17] estimate that 15% of the data collected in a company’s customer databases are erroneous, missing, or incorrect, which all generates erratic data, i.e., databases held by such businesses that are inaccurate or partially lacking sense or cohesion. Overall, some authors have estimated that this could mean added costs as high as close to 10% of some company profits [18], and other authors aimed to measure the general average financial impact of poor data on businesses up to $9.7 million per year, though it is complex to give an accurate estimate nowadays as it is a problem raising during the last years after the increase of digital stored data [19]. In this scenario, data quality refers to the degree of accuracy, accessibility, relevance, timeliness, and data integrity [20,21]. The impact of data quality is crucial for companies and a key factor in the successful implementation of a CRM system [22], as well as one of the biggest challenges [23]. Despite this, it has been pointed out as one of the most underestimated issues in the implementation of CRM [18,24,25,26,27]. The data quality does not only depend on accurate data introduction in the system, but it is also affected by the way the data are stored, managed, and shared [28]. Among the main challenges in this area, we can consider the existence of duplicated entries. As the amount of data grows exponentially, the possibility of having redundant data notably increases, which in some cases cannot be acceptable or affordable, meaning that a preprocessing stage is required. The resolution of entities or the link of records is the task of detecting records that refer to the same entity in different databases. This task has many applications in different fields of research, and for this reason, different terms have been adopted for the same concept, such as duplicate detection, deduplication, reference matching, object identification, combination and purge, object consolidation, or reference conciliation. Entities mostly commonly correspond to people (for example, patients, clients, taxpayers, and travellers), companies, consumer products, or publications and appointments, among others [29]. In this work we use the term *duplicate* to broadly refer to this data-quality problem.

One origin of data duplicity that can be pointed out is the lexical heterogeneity that occurs when the databases use different representations to refer to the same real-world object [30]. For example, for a hotel chain with a large number of hotels that use management programs or Property Management Systems (PMS), the fusion of multiple data sources from these programs may be very complicated. In this scenario, a chain hotel can enter a customer’s data with a customer identification number (ID) that is slightly different from a previous record that was already entered in the PMS, so that several records can be created for the same client, and this could increase rapidly through the different hotels within the chain. On the other hand, common names [31], misspellings, nicknames or abbreviated names, phone numbers, and addresses can be particularly troublesome to be standardized, especially when it comes to guest data from many countries. For example, and according to Revinate (an international CRM technological firm) [32], considering a chain with 50,000 profiles, the combinations in the introduction of customer data can originate up to 2.5 billion records of profiles to be evaluated. Different problems can result from the presence of errors in the data collection, namely, hotels may not recognize repeating guests, duplicate communications could be sent to the same person, or even the expense or the nights could be not correctly calculated. In short, duplicities can lead to erroneous decisions by the chain that affect its profitability and its brand. Although the problem of duplicities is a partly avoidable organizational problem, for instance by having data collection rules for all employees in the chain (and most chains already have) [28], a question arises: What happens to the data that were collected before the implementation of these rules? Moreover, what happens if even after implementing these rules, mistakes still occur in the data collection? It cannot be assumed that the receptionist or booker will have access to the duplicate records, but the fundamental problem is that, in many cases, there are duplicate records that the CRM does not detect. It has been estimated that near 2% of contact data can go bad each month, which is almost a quarter of the database annually [33]. For all these reasons, computer programs are necessary to detect and eliminate duplicities.

On the other hand, there are different tools for data cleaning based on different criteria, such as individual fields or entire documents. In this paper, we focus on finding duplicated customers from the analysis of the individual fields of the CRM entry coming from lexical heterogeneity. Some authors analyze duplication manually by detecting variations in the values of certain variables (such as city or company) in a customer database [34]. Other authors have used different techniques based on character-based similarity metrics for matching fields with string data [30]. Some examples are the Edit Distance [35], the Affine Gap Distance [36], the Smith-Waterman Distance [37], or the Jaro Distance [38], among others. In this work, we focus on the possibilities to find duplicated fields in a hotel-chain CRM by using the Edit Distance, which is built according to the general idea that two values in a string field are duplicated when, by only changing a reduced number of characters, we can transform one value into another [39]. Specifically, we propose using one of the most popular Edit Distance algorithms, called the Levenshtein distance [35], to address duplicated-customer identification as a large-scale data analysis in the CRM from a multinational hospitality chain in Europe, with more than 300 hotels. To support the implementation of comparison strategies, a big-data implementation using map-reduce is used. Aiming to base it on a principled approach to generate efficient yet computationally affordable rules, the information conveyed by these distances was scrutinized in terms of their statistical properties. In particular, the entropy and the mutual information [40,41] of the fields in the CRM forms were analyzed with intensive calculations to show that convergence in their estimation can be readily obtained. Overall, these previous findings suggested creating an *X-from-M* criterion to reduce the computational complexity of the search algorithm and to estimate possible neighbours to a duplicated-customer candidate, hence providing the managers with a moderate number of candidates.

The rest of the paper is organized as follows. In Section 2, we summarize some existing literature related to the duplicate identification whose fundamentals have been useful for our proposal. In Section 3, the detection system for duplicated recordings is presented, together with the required notation and the statistical related concepts for its analysis. Then, Section 4 presents the obtained results throughout all the system elements. Section 5 discusses the significance of the results and the implications of the use of big data in the current CRM and hospitality industry scenarios, together with the statement of final conclusions.

## 2. Background

Managing all the available information in a company and ensuring that this information is of the highest-possible quality means that the value of this company grows significantly [42]. In this setting, the problem of Entity Reconciliation (ER) is one of the fundamentals in the integration of data. As noted above, this problem is also known as deduplication, conciliation of references, purge, and others. ER is a significant and common data cleaning problem, and it consists of detecting data duplicate representations for the same external entities, and merging them into single representations [43]. This problem can be applied to many different domains, such as deduplication in databases [44], duplicate detection in xml data or hierarchical data [45], cross-document co-reference resolution methods and tools [46], blocking techniques [43,47], bug reports [48], customer recognition [31], and E-health [49]. Most of the existing studies have been validated using real-world datasets, but very few of them have applied their proposal in a real case in the industry [42]. In the literature, the duplication detection problem has been studied from three distinct analysis paradigms, namely, ranking, binary classification, and decision-making problems. Among these three problem types, the ranking problem attracts the most attention because of its feasibility, and text mining plays a crucial role in detecting duplicates [48]. Authors in [31] used Levenshtein Edit Distance for feature selection in combination with weights based on the Inverse Document Frequency (IDF) of terms. Matching dependencies, a new class of semantic constraints for data quality and cleaning, has been shown to be profitably integrated with traditional machine learning methods for developing classification models for ER [43].

As previously discussed, CRM is an important framework for managing an organisation’s interactions with its current and future customers [50,51]. Identification of duplicated customers in the CRM is also receiving increasing attention in the literature [52,53,54,55]. When there are many customers in the CRM database with similar names, or names with similar spellings to those of the customer to be identified, recognition becomes difficult. The current literature on identity recognition focuses on searching for and matching a given identity from all the available information in an organization’s database. A clean database improves performance and subsequently leads to better customer satisfaction [31]. It has also been clearly established that good data quality allows the staff to be more productive, as instead of spending time validating and fixing data errors, they can focus on their core mission [56]. For datasets which are noisy or use different data formats, they have to be normalized by employing data transformations prior to comparison. As comparing entities by a single property is usually not enough to decide whether both entities describe the same real-world object, the linkage rule has to aggregate the similarity of multiple property comparisons using appropriate aggregation functions [57]. An algorithm is presented in that work which combines genetic programming and active learning in order to learn expressive linkage rules which can include data transformations and combine different non-linear similarity measures. The user is only required to perform the much simpler task of confirming or declining a set of link candidates which are actively selected by the learning algorithm to include link candidates that yield a high information gain. However, few of these previous studies have devoted space to analyzing the statistical properties of the data fields usually found in a CRM in order to generate statistically principled and computationally operative methods in this arena.

## 3. Detection System for Duplicated Recordings

A database of customer recordings in a CRM can be seen as a multidimensional data structure, usually corresponding to an SQL structured type. The CRM data processing can often become unmanageable either by the dimensions or by the nature of the data, or by both of them. In order to achieve efficient and scalable data processing, it can be advantageous for instance to adapt the data to a file system (unstructured) or to a database (structured) and using queries on them, and sometimes it even compensates to distribute those data in different nodes. In this way, a map-reduce-based solution can be fruitful in order to extract useful information from the data in an embarrassingly parallel way [58] from all the above data arrangements. In this work, we focus on the result of a query obtained from a universe database and turned into a datastore, in such a way that CRM registers here are stored as a set of features for each recorded customer. We also stress here that there will be relevant and irrelevant interdependence among customers, especially when dealing with international delegations of a hospitality company. Moreover, a good number of customers (mostly the multinational ones) will have themselves a complex structure, in terms of headquarters, delegations, and their inter-dependencies. Overall, if we wanted to consider the problem with a strict-realistic view, we would probably need a graph-based structure, a tree-based structure, or even a combination of both. This is probably one of the main reasons for the moderate attention paid to the data duplication in CRM in recent years, despite the practical relevance of this problem.

### 3.1. Problem Notation

If we denote the set of customers in a CRM as $\left\{C^{n},n=1,\cdots ,N\right\}$, the data structure for each customer can be seen as given by a concatenation of *M* features, denoted as $\left\{F_{m},m=1,\cdots M\right\}$. This represents a strong simplification on the data structure, as we are only working with a single-table view of the features. Nevertheless, several of the identification fields likely include implicit information about those relationships, so that it can be subsequently exploited. On the one hand, what we gain with this problem formulation is a simplification that allows us a first approach to the problem without being obscured by the implicit and explicit complex data structures, in other words, we are turning a multidimensional-database analysis problem into a simpler feature-comparison approach. On the other hand, our database now has form fields with different types. Let us assume that each feature belongs to one type in a set of different possible data types. Three of the most usual feature types are categorical, metric, and free text (denoted here as C,M, and T, respectively), and it is also very usual that the categorical features can be expressed in terms of text chains associated to each category value. Therefore, we can define a property type for each of the previous feature types, this is,

(1) $$F_{m}.type\in \left\{C_{I},C_{T},M,T\right\}$$

where categorical features are either indexed values ($C_{I}$), or each category value is associated to a descriptive text string ($C_{T}$). In general, a categorical feature $F_{m}$ has several possible categorical values, which is generally denoted as $\left\{V_{m}\right\}$ (set of values for the *m*th feature), and the possible values are denoted by $\left\{v_{m}^{1},\cdots ,v_{m}^{r_{m}}\right\}$, with $r_{m}$ the number of possible categorical values for $F_{m}$. It can be seen then that even a simple table can have a complex data structure. Finally, we denote the instance matrix as $C_{n,m}=C_{n}^{m}$, which conveys the recorded values of all the features for all the customers in the form table.

The multidimensional statistical density distribution can be denoted as $f_{C}\left(C\right)$, and it can be seen as the joint probability distribution for each feature, taking into account that they can have their different types, this is,

(2) $$f_{C}\left(C\right)=f_{F_{1},\cdots ,F_{M}}\left(F_{1},\cdots ,F_{M}\right)$$

and hence, matrix C conveys sampled instances from this distribution. Let us assume in addition that we can distinguish two regions, which are statistically modeling two groups of instances in this multidimensional distribution. We denote as the *target group* to that set of instances corresponding to a given same customer, and the remaining are called the *non-target group*. Note that the non-target group can include other customer duplicated, but they are not considered as target in this model, so the targets are considered one each time. Then, the statistical region $G_{0}$ ($G_{1}$) correspond to the support of the sampled instances of non-target (target) groups, given by $G_{0}$ ($G_{1}$) sets. From a practical point of view, $G_{1}$ represents that region yielding the sampled set of CRM duplicated instances which the manager wants to identify. From a theoretical point of view, the data structure presents several limitations in different aspects which make their identification hard to address. First, we do not know the labels for the instances in both groups, hence this is a non-supervised problem in Statistical Learning Theory. Second, a method for the comparison among different features is not easy to define, given their categorical and in general heterogeneous nature. Third, even the comparison among instances of the values in the same fields is twofold complex, because we do not have a clear pattern to identify duplicated entries, and we do not either have theoretical knowledge about their statistical distributions.

### 3.2. Distance Distributions in Heterogeneous Recordings

To overcome these challenges, we state the practical problem of CRM duplicated-customer identification as follows. We select a specific test instance $C_{t}$, which is a candidate to be scrutinized for the presence of its duplicates. The managerial criterion to select this test instance can be because it represents a relevant customer, or it is just a new customer to be introduced into the CRM and to be checked in advance that it is not in it. According to the previously described statistical formulation, $C_{t}\in G_{1}$. Our operative problem is now stated as the identification of other possible instances of $G_{1}$ in the database. A condition that can be established to design an appropriate algorithm is that it needs to be more sensitive (emphasis is on not excluding true duplicated instances) even if it represents decreased specificity (a larger number of non-duplicated customers are included as duplicate candidates). This is practically acceptable because the system aims to select a moderate set of candidates which can be subsequently scrutinized by the managerial team, hence connecting the automated tasks and the domain knowledge by the expert users.

In order to tackle the inter-feature issues, we initially assume independence among features, so that their statistical joint distribution can be expressed as the product of the individual feature distributions, i.e.,

(3) $$f_{C}\left(C\right)≃f_{F_{1}}\left(F_{1}\right)\cdot f_{F_{2}}\left(F_{2}\right)\cdot \ldots \cdot f_{F_{M}}\left(F_{M}\right)$$

For an operative design of the intra-feature analysis, we distinguish both groups, i.e.,

(4) $$f_{C}\left(C|G_{k}\right)≃f_{F_{1}}\left(F_{1}|G_{k}\right)\cdot f_{F_{2}}\left(F_{2}|G_{k}\right)\cdot \ldots \cdot f_{F_{M}}\left(F_{M}|G_{k}\right)$$

for k=0,1, and where each conditional distribution has its own shape and parameters. Let us assume now that we can use a distance between two values of a given feature in two customers $n_{a}$ and $n_{b}$, denoted as $d(C_{n_{a}}^{m},C_{n_{b}}^{m})$. The statistical distribution of this distance for the *m*th feature is a random variable, with distribution

(5) $$f_{d^{m}}\left(d^{m}\right)=\frac{1}{s}E_{G}\left(d(C_{n_{a}}^{m},C_{n_{b}}^{m})\right)$$

where *E* denotes statistical averaging and *S* is a normalization constant to unit area. In terms of this distribution, we can say that a customer is a possible neighbor of the test customer if their distance is small.In statistical terms, this can be given by its distance belonging to the threshold distance corresponding to a low percentile $\alpha_{m}$ of the distribution, this is, $d\left(C_{n_{0}}^{m},C_{n_{1}}^{m}\right)<d_{\alpha_{m}}$. We call $d_{\alpha_{m}}$ the threshold distance for *m*th feature to consider two instances as neighbors in that feature.

A remarkably useful distance suitable for strings is the Wagner-Fischer algorithm to compute the Edit Distance between two string characters. This algorithm is extremely simple, and it has a history of multiple inventions [59,60,61]. As seen in the introduction, the Edit Distance determines how dissimilar two strings are to each other by counting the minimum number of operations that are needed to transform one of them into the other. Different definitions of an Edit Distance use different string operations.One of the most popular ones is the Levenshtein distance, in which the admitted operations are the deletion, the insertion, and the substitution of a single character in the string at each step. In the Wagner-Fischer algorithm, the Edit Distance between two strings $a=a_{1}a_{2}\cdots a_{A}$ and $b=b_{1}b_{2}\cdots b_{B}$, is given by the following $d_{i,j}$ recurrence:

(6) $$\begin{array}{ccc} d_{i0} & = & \sum_{k=1}^{i}w_{del}\left(b_{k}\right),\;\mathrm{for}\;1\leq i\leq A \end{array}$$

(7) $$\begin{array}{ccc} d_{0j} & = & \sum_{k=1}^{j}w_{ins}\left(b_{k}\right),\;\mathrm{for}\;1\leq j\leq B\\ d_{ij} & = & \left\{\begin{array}{cc} d_{i-1,j-1}, & \;\mathrm{for}\;a_{j}=b_{i}\\ \min\left\{\begin{array}{c} d_{i-1,j}+w_{del}\left(b_{i}\right)\\ d_{i,j-1}+w_{ins}\left(a_{j}\right)\\ d_{i-1,j-1}+w_{sub}\left(a_{j},b_{i}\right) \end{array}\right\} & \;\mathrm{for}\;a_{j}=b_{i} \end{array}\right\},\;\mathrm{for}\;1\leq i\leq A,1\leq j\leq B \end{array}$$

which is the most basic form of this algorithm and can be readily programmed [60], and where $w_{del}$ ($w_{ins},w_{sub}$) denotes the number of character deletions (insertions, substitutions). This distance provides us with a method to characterize the neighbourhood of test customer $C_{t}$ to detect its possible duplicated customers among its neighbors. Note that we need to identify distributions $f_{d^{m}}\left(d^{m}\right)$ and to define the set of thresholds $\left\{d_{m}\right\}$, which can be either established according to the manager experience when convenient, or with a statistically supported percentile $\alpha_{m}$. In the following we describe the basic principles subsequently used to establish the multidimensional criterion according to the statistical properties of these distributions.

### 3.3. Entropy and Mutual Information Analysis of Distances

The most fundamental concept of Information Theory is the entropy, which characterizes the uncertainty within a random discrete variable. Whereas it was initially introduced in the telecommunication field, it has been subsequently expanded to a wide range of discrete statistics and its applications. Based on it, the mutual information informs us about the amount of information that a random discrete variable contains about another, which is sometimes also described as the reduction in the uncertainty of one variable thanks to the knowledge of the other one [40,41].

In this work, we study the entropy within a given field of a CRM form and the mutual information among the different fields with text type content. We restrict ourselves to a unified and simplified study, in which the fields can be represented one way or another by a not-too-long text. Let $f_{d^{m}}\left(d^{m}\right)$ and $f_{d^{n}}\left(d^{n}\right)$ be the described mass density functions of the *n*-th and *m*-th variables in the CRM form. Then, we define the entropy of the *m*-th variable as follows

(8) $$H\left(m\right)=-\sum_{d^{m}}f\left(d^{m}\right)log_{2}\left(f\left(d^{m}\right)\right)$$

where the use of the base-2 logarithm implies that its units are bits. Accordingly, the mutual information between the *n*-th and *m*-th variables is given by

(9) $$I\left(m,n\right)=\sum_{d^{m},d^{n}}f_{d^{m},d^{n}}\left(d^{m},d^{n}\right)\log_{2}\left(\frac{f_{d^{m},d^{n}}\left(d^{m},d^{n}\right)f\left(d^{m}\right)f\left(d^{n}\right)}{f_{d^{m}}\left(d^{m}\right)f_{d^{n}}\left(d^{n}\right)}\right)=E_{d^{m},d^{n}}\left(\frac{f_{d^{m},d^{n})}}{f_{d^{m}}f_{d^{n}}}\right)$$

Therefore, in our problem entropy represents the amount of information of each of the variables in terms of the mass density of the Edit Distances among its instances. On the other hand, the mutual information represents the amount of information shared between two variables in terms of the same Edit Distance. Both represent a basic but fundamental statistical description to begin to analyze the distributions of distances in these abstract spaces of text strings, and they will allow us to scrutinize a principled way to adjust thresholds in the heuristic approaches to our problem.

### 3.4. Dealing Joint Distributions with X from M

The remaining issue to deal with is the inter-feature dependencies, which is really complex. For this purpose, and supported by the experiments related with the previous section, we propose here implementing the criterion so-called *X from M*. This means that, from the *M* available features, we consider that an instance is a *global neighbour* of $C_{t}$ if at least *X* features are at a distance below their threshold, independently of which features they are. This represents a robust yet simple criterion, which aims to give a tool to drive the search with flexibility of the origin of the duplication in the database.

### 3.5. Map-Reduce Implementation

Even with the above described simplifications on the data structure and on the statistical distributions, the amount of data to deal with in a CRM (several hundred thousands or often millions of entries) can be a strong limitation for any algorithm. In this scenario, as in many others, big-data techniques can be useful to solve serious and practical problems such as disk memory problems, random-access-memory problems or computational cost problems, making the problem unmanageable. These problems are mainly caused by the computational cost of the used algorithms and by the size of the data. Depending on the limitations of the problem, the most appropriate option among a variety of existing big-data techniques can be chosen [58]. One of the most popular methods is map-reduce, which allows division of the data and process them among different nodes and calculate the solution in an embarrassingly parallel way. Map-reduce divides the problem into different chunks, which could already be divided if the database or file system was previously distributed, and dispensed in different nodes (workers). These workers apply a function called map (mappers), whose operation must be commutative and associative and the obtained values are grouped according to the different existing required keys. This is known as a key-value paradigm. Once the values have been grouped by key in each node, all of these lists of values of a certain key can be reduced to a single value by means of a function called reduce. This function is executed by new assigned workers (reducers) who receive as input all of these lists of values assigned to a particular key.

In our work, map-reduce implementations were used basically to parallelize those tasks involved in running neighbor comparisons throughout the large database, which mostly involved the neighborhood loops, and the experimental estimations of mass density functions and probability density functions. Nevertheless, given their repetitive nature in the neighborhood comparison processes, it is evident that big-data technologies can be especially useful in order to support the proposed application, even with the use of basic tools available in them.

## 4. Experiments and Results

### 4.1. Database Description

Our method was built while using it on a real data problem, and a database was assembled from the CRM universe of a large hospitality company. It consisted of more than 800,000 recordings in a recovered table, in such a way that each customer was included in a row, and M=18 features were included in text form, either categorical, integers, or text strings. In other words, the feature formats were of such a different nature as numerical integer identifiers, nominal identifiers (names), segmentation-categorical fields, localization fields, and binary values. These recordings corresponded to the hospitality-company data recorded during years 2016 and 2017.

We scrutinized and quantified the different theoretical elements and simplifications that have been described in Section 3. Note that those features containing names (nominal identifiers) sometimes can be optional and free-text input, such as names of people who are responsible for a given environment. Other kinds of variables with different a nature are those features related to segmentation or geographical localization, which can represent very different aspects, but they are often categorical features with a predetermined number of categories, and this number is often reduced compared to the number of recordings. This represents a different situation compared to nominal variables with free text, whose number of classes could be comparable to the number of recordings. Nevertheless, the same distance can be used for them with operative advantage, as shown in the following experiments.

### 4.2. Intra-feature Distance Distributions

We started by analyzing the distribution of the intra-feature distances for each of them individually. For this purpose, a customer test $C_{t}$ was randomly selected and compared with a subset of other 100,000 customers, $\{C_{n^{\prime }},n^{\prime }=1,\cdots ,$100,000}, which was repeated for 100 independent realizations. Our comparison consisted of calculating distance $d(C_{t}^{m},C_{n^{\prime }}^{m})$ for each m=1,⋯,M feature, with $n^{\prime }=1,\cdots ,$100,000, i.e., we obtained the distances from the test customer to all the other ones for every feature. This allowed us to build an estimation of the empirical statistical distribution in terms of their histograms.

**Figure 1:**
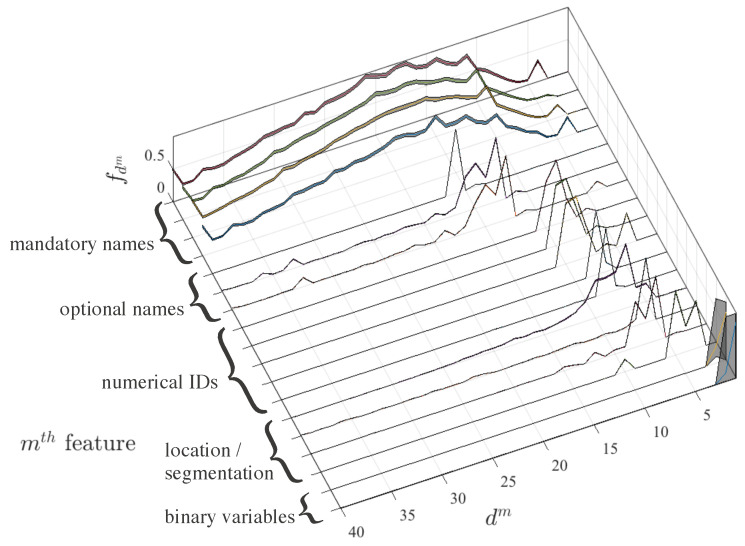
Normalized $f_{d^{m}}\left(d^{m}\right)$ for the different features in the experimental database. Features have been sorted in descending order of the position of the maximum of each distribution, for visualization purposes, which is strongly consistent with their nature, as seen.

Figure 1 shows the averaged estimated distributions for these intra-feature distances for each *m*th feature. For visualization purposes, distributions $f_{d^{m}}$ are represented as normalized to their maximum value. The shaded area represents their 95% confidence interval. It is noticeable that this confidence interval is very narrow in practically all the variables, which is due to the facts that distributions are strongly discretized, and that a large number of examples is used for their construction. Hence, even the peaks in the fragmented distributions are not attributed to noise, but rather consistent among realizations. Note also that the distributions have been sorted in terms of descending position of their absolute maximum, and this in turn makes them group together in reference to their nature. Hence, the most widespread distributions correspond to mandatory names, which are free-text string characters. These distributions have a region of short distances (about less than 5), a single-modal distance and a tail (which is not shown as the trimmed histogram has been represented for visualization purposes). Then, features related with optional names exhibit narrower distribution mass, some tails, and short distances being around less than 10.

A similar pattern is followed then by numerical identifiers and location/segmentation variables, whose profile is in general narrow, with tails and with lower short distance. Obviously, binary features have the shortest distributions. These results point out that it seems feasible to use the same distance with all of these features, even despite their different nature, because neighborhood can be defined with a similar criterion. On the other hand, the neighborhood distance is to be defined for each feature, but it seems to be consistent with features of the same kind, which can guide their assignment, either from an automated or from a supervised approach.

### 4.3. Tails of the Generalized Edit Distance

We subsequently analyzed the dependence of the Edit Distance with the percentile. Note that in the problem of duplicated customer identification, as explained before, distances larger than 5 or 10 characters, depending on the feature, could yield completely different strings, independently of their length.

**Figure 2:**
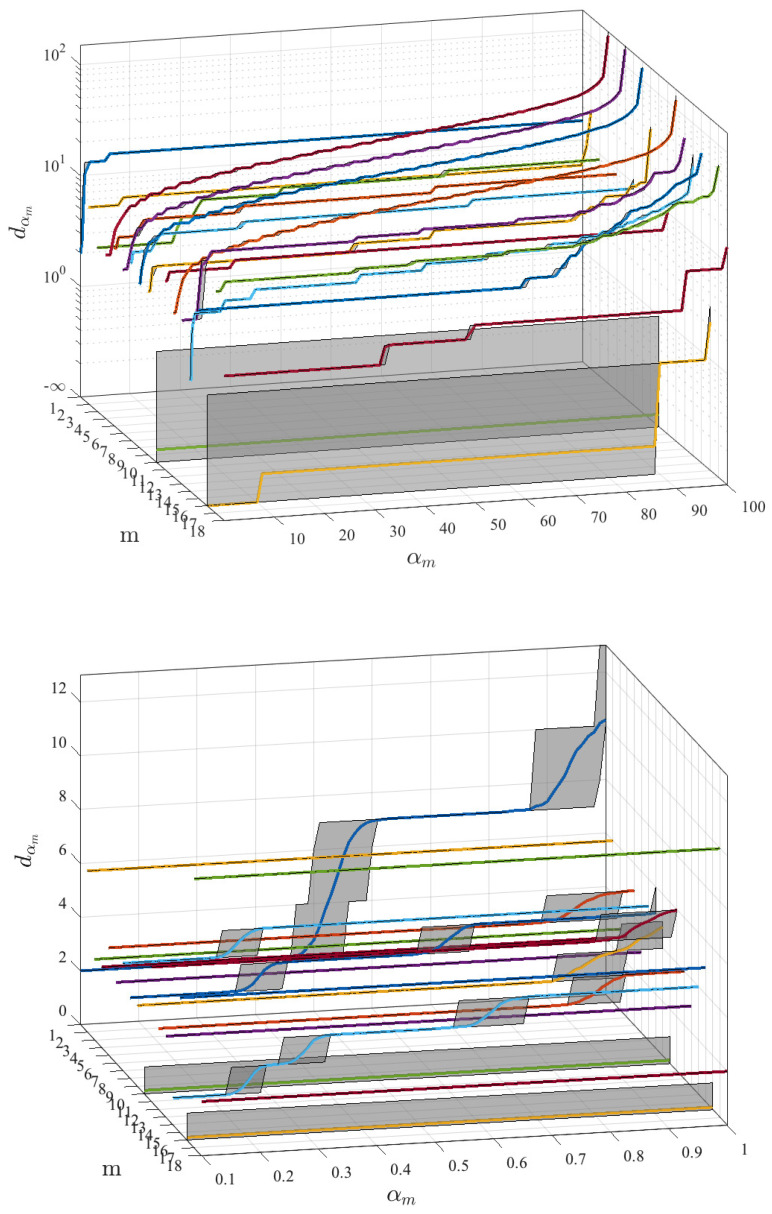
Distance in number of characters obtained for $d_{\alpha_{m}}$ when screening percentile $\alpha_{m}$ in the statistical distributions of each of the *m* features. Panels show mean and 95% confidence interval (shaded in gray) for the 100 independent realizations. Panel down is a zoom for the range $\alpha_{m}<1$, given that this region turns to be the most interesting one for neighborhood purposes of distance $d_{\alpha_{m}}$.

Figure 2 shows the representation and detail for lower percentiles of this relationship in the performed experiments. In order to determine the distance of interest, percentiles lower than 1% should be used in this problem and in this database. The lower panel shows the distance for $\alpha_{m}<1$ for each of the *m* features, including averaged value and the 95% confidence interval. These representation show a strong step-like aspect, with wide confidence intervals in the transitions, which suggests that the use of $\alpha_{m}$ percentile as a threshold could turn unstable and even inefficient, because a range of percentiles will result in the same neighborhood distance. Therefore, we propose to use the distance value $d_{t}^{m}$ as threshold, in number of characters or simple operations, as the threshold value to be fixed by managers using the system.

Nevertheless, and after these observations, further analysis of the text vector spaces induced by the Edit Distance can be done in terms of the asymptotic properties of the entropy and the mutual information of the variables, which in turn can be useful to design principled data quality algorithms in CRM environments, as introduced next.

### 4.4. Entropy Analysis of Text Features in Edit-Distance Induced Spaces

To characterize the fundamental properties of the text spaces induced by the Edit Distance in data quality applications in CRM, the following set of experiments was carried out. First, for a given length of available data (*N*), we randomly selected data from the total CRM, and the mass density function of each variable used was estimated within this subset. This experiment was repeated 100 times by taking random samples from the database with also randomized initialization, and the 95% CI of said estimation was calculated. In addition, the asymptotic behavior of the CI was studied by increasing the value of *N*. Secondly, considering the same data with which the estimates of the mass densities were calculated, the entropy of each characteristic and of the mutual information of all pairs was estimated. In the same way, its asymptotic evolution with *N* was also analyzed.

**Figure 3:**
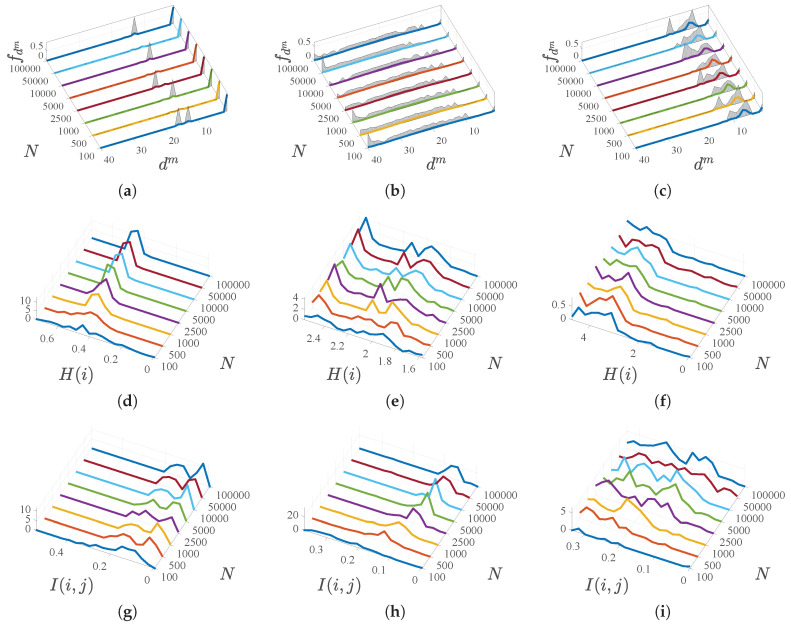
Density mass function estimations and their asymptotic CI for examples of features with different nature (**a**–**c**). Estimated entropies and asymptotic evolution for representative example features (**d**–**f**). Estimated mutual information and asymptotic evolution for representative examples of pairs of features (**g**–**i**).

Figure 3 shows representative examples of these results. In particular, the figure represents in each panel (a,b,c) the estimated density mass function and their corresponding asymptotic CI for examples of features with different nature as a function of the distance for each of them, and calculated of a different set of compared samples *N*. Note that the densities are amplitude normalized for better visualization. The (non-amplitude-normalized) density distribution of the estimated entropies are represented in (d,e,f) for three different examples of representative types of features, and their asymptotic evolution can be observed when increasing *N*, which in general can be appreciated to be mostly stabilized for N>1000. Also the figure represents the (non-amplitude-normalized) density distributions of the estimated mutual information and asymptotic evolution for representative examples of pairs of features (g,h,i).

It can also be seen in that figure that the mass densities of each characteristic have different profiles, and they are strongly multimodal, see panels (a,b,c). In all of them there is a gap between what we could describe as being very close and being further away. However, the length of this gap varies in different variables, as it does the width of the single mode or several modes corresponding to separated distances. It is of great practical interest to see that mass densities are estimated accurately for each variable without the need to asymptotically rise to extremely high values of *N*, which supports the feasibility of estimating these densities if needed by probabilistic methods.

On the other hand, and as a result of the different profiles of the mass densities, the entropy of the different variables also turns out to be sometimes multimodal, though not always, see panels (d,e,f)). Note that the entropy values are moderate (of the order of magnitude of some few bits, and less than one bit for the special case of categorical variables). Regarding mutual information, some bimodalities can be seen in panels (g,h,i), though they are not excessively relevant because in any case they have low values, usually less than a bit. This means that the information provided by each of these random variables about the others is low, therefore, this indicates a low reduction of uncertainty and low dependence among them. Again, the estimation consistency is in general quickly reached for the entropy and for the mutual information, meaning that more advanced probabilistic methods could be used in this scenario.

### 4.5. Distance Increase with Multidimensional Spaces

Another relevant aspect which needs to be quantitatively analyzed is how restrictive can a duplicated search be, as a function of the number of features that have to be below their fixed threshold $d_{t}^{m}$. Based on the results of the previous experiments, we propose the following approach. If we focus only on some more apparently attractive-to-use features we could be missing profiles from features a priori less interesting, but which could be revealed as relevant by an automatic search. Therefore, in order to characterize and measure the constrained character of the search, we measured the number of neighbors of $C_{t}$ obtained by screening the number of thresholds to be fulfilled, in the so-called *X from M* criterion, if the distance threshold is fixed $d^{m}$ between 1 and 5 characters. This is consistent with the gap asymptotically determined in Figure 3. From a practical point of view, we restricted ourselves to search up to 5 characters because a larger number was observed to give an extremely large number of candidates to be post-analyzed by an expert.

**Figure 4:**
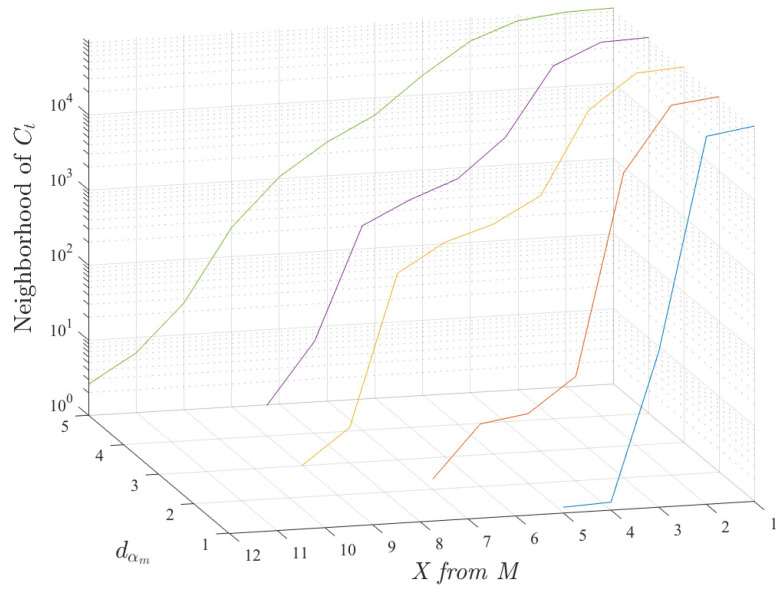
Averaged neighborhood of a specific customer $C_{t}$, obtained for 100 independent realizations, as a function of the fixed threshold in distance $d_{\alpha_{m}}$ and the number of features required to fulfill its corresponding threshold, according to the *X from M* criterion.

Accordingly to the previous considerations, Figure 4 shows the results of calculating the averaged neighborhood of a specific customer $C_{t}$, and repeating the experiment randomly for 100 independent realizations in the same available database. The vertical axis shows the mean size of the neighborhood as a joint function of the fixed threshold in distance $d_{\alpha_{m}}$ and the number of features required to fulfill its corresponding threshold, according to the previous setting of the choice of the value for *X from M* criterion. Note that any possible combination of *X* features has been scrutinized in this experiment, and in our database we used M=18 features. As shown in the figure, and for the M=18 considered features, if we try to allow more than 50% of the features in *X from M* (i.e., X>9) for threshold distances $d_{t}^{m}<5$ it turns out to be too restrictive, and we do not get any candidate. On the other hand, for X=3 from 18 we would get too many candidates. The graph represents the joint evolution of the distance and of the number of neighbor features in the scheme, and their inter-dependence. Note also that fixing the distance threshold to $d_{t}^{m}=1$, very few candidates are obtained, which is a case to avoid due to its extreme probability of loss. This allows us to establish both free parameters so that a very well defined search area is defined for them.

### 4.6. Case Study for Real-World Usefulness

The improvement of the quality of a CRM, and more specifically, the cleaning of duplicates, is a task that is usually carried out not only through distance-among-words criteria but also through business criteria. To our best understanding, and as explained so far in the present work, this is the reason why the application of techniques based on distances among words can be very useful for this purpose, as it can reduce the number of candidates to evaluate with business criteria at a later stage.

**Figure 5:**
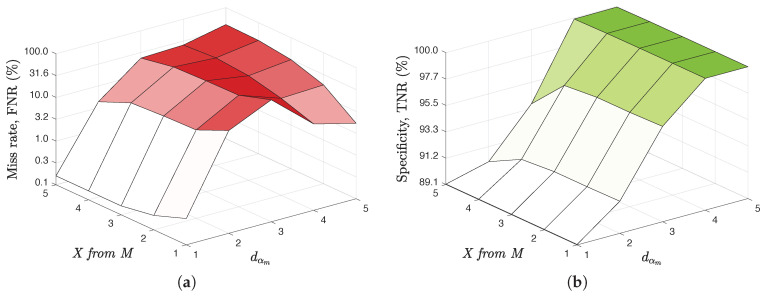
Trade-off between False Negative Rate (**a**) and True Negative Rate (**b**).

In order to further evaluate this usefulness in a simple yet real-case scenario, we applied the technique proposed in this paper on the data of a CRM from a big and international company. Specifically, it was been applied on 3753 duplicates in which the original assignment was known among a total of 363,960 data and in which the possibilities of detection were inside the scope of the techniques proposed here. Following the knowledge offered by the business area, it was applied to 5 of the most critical variables for the detection of duplicates (2 mandatory names, 1 optional name, 1 segmentation variable, and 1 binary variable). Given that it is interesting to offer the lowest number of candidates to a duplicate so that the business criteria can do their refining work, Table 1 shows both the average number of candidates that this technique is capable of saving (i.e., specificity, selectivity, or true negative rate, TNR) and the percentage of times that the original entry to which the duplicate should be assigned has been lost (i.e., missing rate or false negative rate, FNR=1−TPR, being TPR the true positive rate or sensitivity). These measures are given therein for different values of the maximum distance threshold to be set ($d_{\alpha_{m}}\in \left\{1,5\right\}$) and the number of *X from M* variables (being M=5) that would have to meet at least that maximum distance value. Figure 5 shows the trade-off between the missing rate or *100-Sensitivity*, and between the number of candidates correctly discarded or *Specificity* (both in % in the figure).

## 5. Discussion and Conclusions

Several studies [62,63,64] have underlined the relevance of managing customer data at a high quality level.Experian revealed that 66 % of companies lack a coherent, centralized approach to data quality [33]. Loose quality data such as duplicates or missing data, inaccurate or outdated data, could lead managers and analysts to develop an incorrect image of customers and their purchase preferences [65]. This is specially more patent in multinational companies that operate through more channels than in those who operate in a single country. Moreover, the information collected across various channels is frequently exposed to human error as consumers and individual employees enter information manually, e.g., receptionist at the front office. Some authors point out the lack of vision of the organization to share, maintain, use, and improve customer information [66,67,68]. Despite its relevance [69], there is no recognized and efficient data management model to handle big-data (large volume, complex, growing datasets with multiple autonomous sources) since the traditional data models are not able to manage its increasing complexity [70].

In this scenario, our proposal is a big-data system to address the problem statement of duplicated customer identification as a large-scale data analysis in a multinational hotel chain. Duplicated records slow down client’s search engines, issues of the invoice configuration, rules of validation of fields, among others, which suppose that the time lapses of reservation management and invoice issuance (at checkout) substantially increase. In many cases, there are duplicated records but the CRM does not detect them. When detected, asking for these data from the customer could overpass the Data Protection Laws. In addition, the identification rules may be different depending on the country. Therefore, the fundamental problem is not the elimination of duplicates itself but their detection. This is a common problem in the hotel industry and it is worthwhile for them to invest in research in order to identify these duplicates, hence gaining in data quality, information homogeneity, improvement in the measurement of bonus of the commercial area (which is critical), and reduction of errors in the application of tariffs, to name only some few advantages. Even with validated data and improved searching, duplicates will inevitably be created due to the nature of human error. Databases should be checked at regular intervals to ensure that no duplicate accounts have been created and to consolidate their conveyed information. Our approach has consisted of first scrutinizing this specific data quality problem, then providing a statistical problem statement, and finally using statistical simplifications on the data structure to be handled. Even so, the number of operations is large, as far as a CRM in a big company will typically include hundreds of thousands of recordings, hence big-data simple solutions (map-reduce or data stores) can be applied when and for what they are actually needed.

Our method has implemented several simple, yet powerful elements. First, a generic feature representation for the customer fields is given by a table data structure, which can be handled as a heterogeneous data matrix, which has simplified the issue of data heterogeneity. Second, homogeneity in the comparison procedure has been done by working with the different data types in terms of the same distance, namely the Edit Distance, which has been shown in the experiments to be operative enough. The differences in the intrinsic neighbourhoods of potentially duplicated recordings tend to be similar among features of the same nature, and somehow different with respect to features with somehow different nature. Nevertheless, the characteristic distances for all of them are always small (we could say less than 10, often much less), in terms of the statistical distribution tail. Third, the use of big-data techniques has been tailored to the needs of this search, hence, the advantage of big data does not come from complex algorithms being parallelized for a given task, but rather it comes from the aggregation effect of a large number of recordings, as shown by the smooth confidence intervals obtained for the estimated distance distributions in our experiments. The estimation consistency was in general quickly reached for the mass density functions of the variables, for their entropy and for their mutual information, meaning that more advanced probabilistic methods could be used in this scenario. Also, the mutual information among variables was extremely moderate, though not null, which needs to be taken into account to further explore statistically principled methods in this scenario. Finally, the previous over-simplification could be expected to preclude the system to efficiently find diverse neighbours, for only working with close individual features. However, it has been shown how the use of a *X from M* simple strategy brings back to the algorithm the power and flexibility of searching for neighbours which could be at unexpected features.

It is very complex to create a completely automatic system, so that a reasonable choice in this kind of scenarios is to create a semi-supervised approach. On the one hand, this strategy can be checked in the literature to be a suitable approach to big data problems in emerging areas, in which data quality is revealed in those early stages to have an even more-than-suspected potential high impact on the company [71,72,73,74,75,76,77,78,79]. On the other hand, the Information Technology areas or similar ones often have staff partially devoted and responsible for the data quality aspects. Nevertheless, we only addressed one single aspect of data quality, which was the customer duplication in a multinational hotel chain CRM, considering that duplicated data can be situated among the top three data quality errors for 30% of organizations [33]. Although the usefulness of high data quality is desirable and clear, in the case of very large databases, this objective may be in conflict with high associated costs. A cost-benefit model [80] confirmed the assumption that the optimal level of data quality, in terms of maximizing net benefits, is not necessarily the highest technically possible. Nevertheless, the study by [18] showed that there is a clear similarity between the data quality factors that affect small and medium enterprises and large organizations from the point of view of CRM implementation. This desirable implantation often shocks [24] with the absence of planning in the stages of data cleaning, normalization, integration or migration, hence data governance has been proposed, which consists of defining a set of policies and procedures to supervise data use and management, and their turning into a strategic active.

Our method is being used in this setting, and the design has been done to provide the manager with increased sensitivity at the expense of moderately reduced specificity when identifying candidates to be duplicated. However, our experiments have shown that the workload for an expert supervisor after our system output is affordable. There are two scenarios that can be established for the proposed system. On the one hand, the manager can be willing to identify the possible duplicated recordings of a given customer. This can be due to this customer being strategically or economically relevant, or just the inclusion of the new customers of every month in the system. Here, a data store solution is enough to perform the analysis and to provide an operative candidate list. On the other hand, a large-scale exploration of the database can be required, for instance, for migration purposes. In this case, the use of more computationally intensive implementations (such as map-reduce, but also any improved state-of-the-art platform) can help to overcome the algorithmic load, while still being operative and usable.

We can conclude that the use of big-data technology can provide hospitality management, and hence many other sectors, with a tool for data quality control and improvement of their CRM systems. Specifically, duplicated customers can be identified, with high sensitivity and operative specificity, with the help of a particularly designed system. The use of simple algorithms and statistical models is compensated by the strength of using large numbers of recordings, which has been shown to provide stable statistical descriptions. Further open issues of big-data and data quality in CRM can be addressed starting from the results obtained here, and their use is expected to grow naturally when the specific challenges are identified through the cooperation between academia and the hospitality industry in this area.

## Figures and Tables

**Table 1:** Trade-off between Sensitivity and Specificity.

	Sensitivity, TPR (%)					Specificity, TNR (%)				
$d_{\alpha_{m}}$	1 from 5	2 from 5	3 from 5	4 from 5	5 from 5	1 from 5	2 from 5	3 from 5	4 from 5	5 from 5
1	89.16	91.66	96.86	99.99	100.0	99.60	77.48	38.08	90.54	94.67
2	89.16	91.61	96.77	99.99	99.99	99.79	89.48	64.19	83.40	81.69
3	89.16	91.61	96.77	99.99	99.99	99.84	90.03	65.28	75.27	68.51
4	89.16	91.39	96.50	99.97	99.99	99.84	90.99	67.39	71.30	60.59
5	89.16	90.00	93.71	99.90	99.94	99.84	95.66	76.37	74.37	58.89
